# miR-92 Regulates the Proliferation, Migration, Invasion and Apoptosis of Glioma Cells by Targeting Neogenin

**DOI:** 10.1515/med-2020-0040

**Published:** 2020-04-08

**Authors:** Yi Wang, Yaohui Tian, Zonghao Li, Zhaoke Zheng, Liangliang Zhu

**Affiliations:** 1 The Second Department of Neurosurgery, the Cangzhou Central Hospital, No. 16 Xinhua West Road, Cangzhou 060000, Hebei, China

**Keywords:** Glioma, miR-92, neogenin, proliferation, migration, apoptosis

## Abstract

This study aimed to explore the pathological mechanism in regulating glioma progression. The expression of miR-92 and neogenin was evaluated by qRT-PCR and western blot. Cell viability and apoptosis were measured by MTT and flow cytometry assays, respectively. The migration and invasion abilities were examined by transwell assays. The interaction between miR-92 and neogenin was conducted by dual-luciferase reporter system. As a result, we found that the expression of miR-92 was up-regulated in glioma tissues and cell lines. Down-regulation of miR-92 inhibited glioma cell proliferation, migration, invasion and promoted cell apoptosis rate of U251 and U87 cells. Notably, miR-92 was identified to directly target to 3’-UTR of neogenin. Furthermore, neogenin was down-regulated in glioma tissues and cells in a miR-92-correlated manner. Overexpression of neigenin could cause similar results to miR-92 knockdown in U251 and U87 cells. However, the silencing of neogenin partially reversed the effects of miR-92 knockdown on cell proliferation, migration, invasion and apoptosis of glioma cells in vitro. In conclusion, we clarified that miR-92 knockdown could suppress the malignant progression of glioma cells in vitro by targeting neogenin. Therefore, miR-92 could serve as a potential diagnostic and prognostic marker in glioma patients

## 1 Introduction

Glioma is one of the most aggressive malignant tumors occurring in the central nervous system with high mortality, and accounts for over half of brain tumors [1, 2]. Conventional treatments for glioma include surgery, chemotherapy and radiotherapy; however, the poor outcome and high recurrence make glioma a global challenge that threatens human life and well-being [3, 4, 5]. Therefore, research on the molecular mechanism of glioma origination and progression is urgently needed, and could provide potential prognostic and therapeutic targets.

MicroRNAs (miRNAs), small non-coding RNA molecules with 18-23 nucleotides, act as either oncogenes or suppressors to mediate post-transcriptional gene silencing, and to further regulate tumor progression by binding to their target genes [6, 7, 8]. For instance, the overexpression of miRNA (miR)-20a was reported to promote cell proliferation in anaplastic thyroid cancer by targeting CELF2 [9]. Meanwhile, miR-139 was low-expressed, and inhibited liver cell growth and metastasis through targeting CXCR4 [10]. miR-92, one member of the widely-known oncogenic miR-17-92 cluster, has been identified to promote cell proliferation in many tumors, like lymphoma, breast cancer and lung cancer [11]. Ada Sacchi’s group also discovered that miR-92 increased myeloid cell proliferation and inhibited cell death by targeting p63 abundance and modulating isoforms [12]. However, the biological role and regulatory mechanism of miR-92 in glioma remain largely elusive.

Neogenin, receptor of various ligands (netrins, secreted protein, bone morphogenetic proteins, etc.), functions in neuropathic detection, ion mediation and embryonic development regulation [13, 14, 15]. Notably, neogenin is involved in multiple brain diseases, like Alzheimer’s disease and glioma, since it participates in cellular axon guidance, trophectoderm differentiation and neuronal regulation [16, 17]. Moreover, neogenin has the capacity to active cell death pathway and modulate cell migration through the consumption of netrin, thus the dysregulation of neogenin has been reported in many primary tumors, such as squamous cell carcinoma, breast cancer and gloima. Previous reports demonstrated that the low expression of neogenin in glioma induced promoter methylation and accelerated glioma progression [18]. However, it is still unclear whether the regulation of neogenin is executed by binding to miR-92.

In our study, we explored the dysregulation and role of miR-92 and neogenin in glioma cell proliferation, apoptosis, migration and invasion in vitro, as well as confirmed the interaction between both. Furthermore, we clarified a regulatory mechanism of miR-92/neogenin axis in glioma progression, and suggested miR-92 could serve as a potential biomarker for the treatment of glioma.

## 2 Materials and Methods

### 2.1 Tissue samples

A total of 32 primary glioma patients were recruited from the Cangzhou Central Hospital. All the patients did not receive any treatments. Glioma tissue samples and the adjacent healthy tissue samples were collected through surgery and immediately stored at -80 ^o^C for further study. All the volunteers that participated in the study signed informed consents and the study protocols were permitted by Ethics Committee of the Cangzhou Central Hospital

### 2.2 qRT-PCR

Total RNA was extracted from glioma tissue and cell lines by Trizol reagent under the manufacturer’s instructions. RNA was reverse transcripted using an aqMan™ MicroRNA Reverse Transcription Kit and qRT-PCR was performed using Path-ID™ Multiplex One-Step RT-PCR Kit by the standard procedure. The primers for miR-92 and neogenin were listed as follows: neogenin, (Forward, 5’-GAGATGGGGGACTCTACCG-3’; Reverse, 5’-TGTCAAG-CACTTCTTCGTT-3’); miR-92, (Forward, 5’-CCGCGTGCTGG-GATTC-3’; Reverse, 5’-TCCAGAAGGCTGCAAATGG-3’).

### 2.3 Cell transfection

Glioma cell lines U251 and U87 were purchased from ATCC (Manassas, VA, USA) and incubated in 5% CO_2_ incubator at 37^o^C in DMEM medium containing 10% FBS and 0.05% penicillin/streptomycin. Synthetic oligonucleotides or vectors (anti-NC, anti-miR-92, anti-miR-92+siRNA and anti-miR-92+si-neogenin, 50 nM) were transfected into U251 and U87 cells using Lipofectamine 2000 (Invitrogen Carlsbad, CA, USA) following the manufacturer’s instructions. U251 and U87 cells were harvested 48 h post-transfection for subsequent treatments.

### 2.4 Cell viability

Transfected U251 and U87 cells were seeded into a 96-well plate (5000 cells/well) and incubated in a 5% CO_2_ incubator for 24 h, 48 h and 72 h. Then, 10 uL MTT solution (5 mg/mL) was added in each well to react with the living cells. After being washed with PBS 3 times, 200 uL DMSO was added to each well to terminate the reaction. The optical density (OD) values at 490 nm were measured by a microplate reader.

### 2.5 Flow cytometry analysis

Cell apoptosis was analyzed 48 h post-transfection after the cells were co-stained with Annexin V-FITC/ PI using flow cytometry (BD Biosciences, San Jose, CA, USA). Briefly, U251 and U87 cells were stained with 10 uL FITC-tagged Annexin V/PI in the dark for 20 min at 48 h post-transfection. After being rinsed with PBS 3 times, the cells were re-suspended with binding buffer. The apoptosis rate was counted and analyzed by a flow cytometer.

### 2.6 Transwell assay

The migration and invasion ability of U251 and U87 cells were evaluated by transwell assay. The upper chamber was coated with Matrigel (Becton Dickinson, Franklin Lakes, NJ, USA) overnight, then 200 uL transfected cells (2×10^5^ cells/mL) were seeded for another 24 h. Subsequently, the migrated and invaded cells in the lower chamber through the membrane were fixed with 4% paraformaldehyde and stained with 0.1% crystal violet for 10 min, respectively. The stained cells were counted through a microscope (Olympus, Tokyo, Japan) at 200×.

### 2.7 Dual-luciferase reporters assay

The potential binding site between miR-92 and neogenin was predicted by TargetScan. The 3’ UTR sequences of neogenin containing putative wild-type (WT) or mutant (MUT) binding sites of miR-92 were cloned into the luciferase vectors (Promega, Madison, WI, USA) to construct a neogenin-WT or neogenin-MUT vector. Luciferase plasmid and miR-92 mimics were co-transfected into 293T cells using Lipofectamine 2000. The cells were collected at 48 h post-transfection and luciferase activity was evaluated by a dual-luciferase assay system (Promega).

### 2.8 Western blot

Transfected U251 and U87 cells were lysed at 4^o^C with RIPA lysis buffer containing protease inhibitors (Beyotime, Shanghai, China) to extract the total protein. The concentration of the total protein was quantified by a BCA protein assay kit. Equal amounts of proteins were separated on a 10% SDS–PAGE and transferred to PVDF membranes (Millipore, Bedford, MA, USA). After blocking with 5% nonfat milk for 1 h, the membranes were incubated with primary antibodies against neogenin or β-actin (Abcam, Cambridge, MA, USA) at 4^o^C overnight, followed by interacting with a HRP-conjugated secondary antibody for 2h. Neogenin protein was captured and analyzed using Image Lab software (Bio-Rad, Hercules, CA, USA).

### 2.9 Statistical analysis

All the data was analyzed by GraphPad Prism 7 and SPSS statistical software and presented as mean ± standard error (SD) from at least three individual experiments. Student’s t-test and one-way analysis of variance (ANOVA) were applied to analyse the significant differences. Linear correlation between miR-92 and neogenin was calculated by Pearson’s correlation test. P<0.05 were considered as statistically significant.

## 3 Results

### 3.1 Up-regulation of miR-92 expression in glioma tissues and cell lines

**Figure 1:**
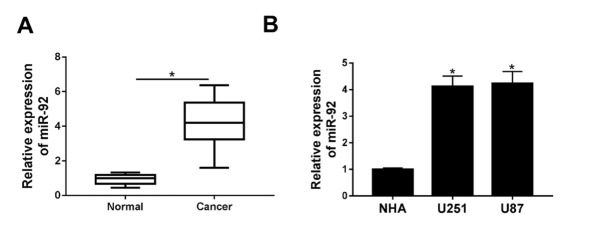
miR-92 was highly expressed in glioma tissues and cell lines. (A) The expression of miR-92 was measured utilizing qRT-PCR assay in 32 pairs of glioma tissues and adjacent normal healthy tissues. (B) qRT-PCR detected miR-92 level in glioma cell lines U251, U87 and normal cell line NHA. *P < 0.05.

The expression of miR-92 in glioma tissues and cells was detected by qRT-PCR prior to the investigation of its role. As shown in Fig.1A, miR-92 expression was remarkably up-regulated in 32 cases of glioma tissues compared with adjacent healthy tissues. Furthermore, we found that miR-92 expression level was higher in U251 and U87 cells in comparison with human astrocyte cells NHA (Fig.1B). These findings suggested miR-92 was highly expressed in glioma, suggesting a potential oncogenic role of miR-92 in glioma progression.

### 3.2 miR-92 knockdown suppressed proliferation, migration, invasion and promoted apoptosis of glioma cells

**Figure 2:**
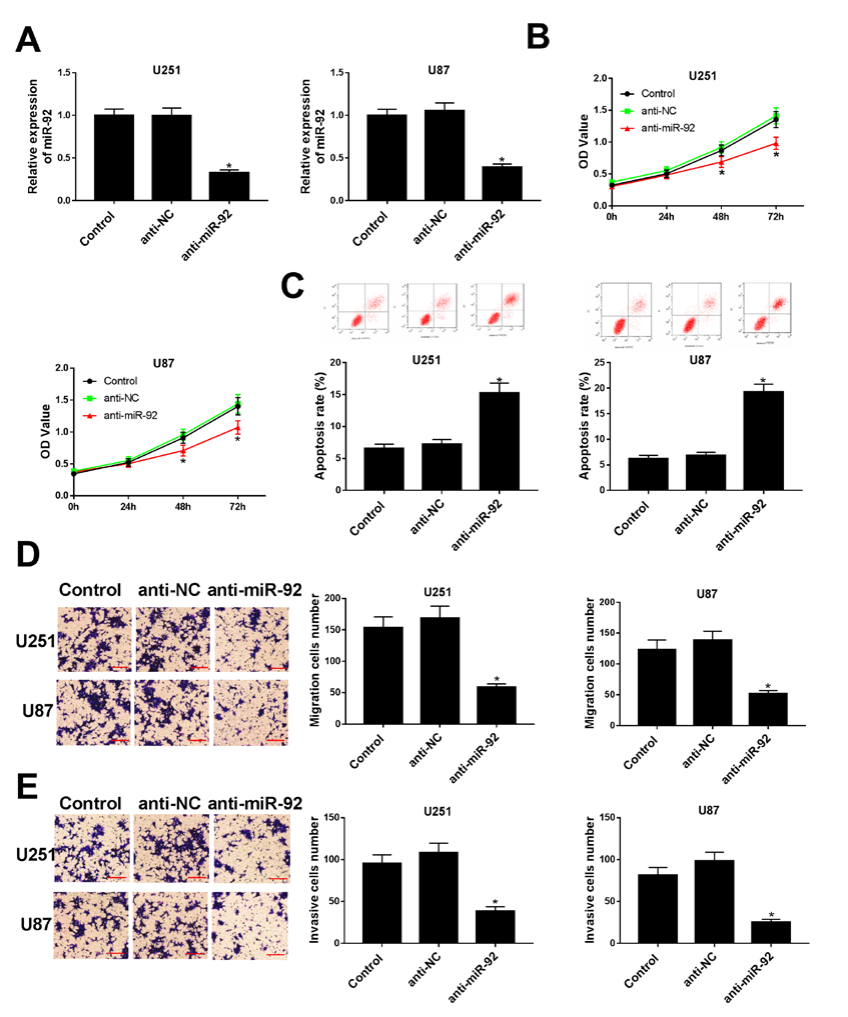
The effects of miR-92 knockdown on the proliferation, migration, invasion and apoptosis of glioma cells. U251 and U87 cell lines were transfected with anti-NC or anti-miR-92. (A) The expression of miR-92 was measured utilizing qRT-PCR assay after transfection. (B) The cell viability was evaluated by MTT assay at different times (24 h, 48 h and 72 h). (C) The cell apoptosis was detected after transfection for 24 h by flow cytometry. (D and E) The migration and invasion abilities were examined after transfection for 24 h by transwell assay. The representative images were presented at 200× magnification. Scale bar: 100 μm. *P < 0.05.

The regulatory effects of miR-92 knockdown on glioma cells progression were verified *in vitro*. Then, U251 and U87 cells were transfected with anti-NC and anti-miR-92 to conduct the subsequent biological function. Compared to anti-NC transfection group, miR-92 expression was decreased after being transfected with anti-miR-92 in both U251 and U87 cells (Fig.2A), indicating that the transfection process was successful. MTT results showed that miR-92 knockdown significantly suppressed glioma cell proliferation at 48 h and 72 h (Fig.2B). In addition, the apoptosis rate of U251 and U87 cells transfected with anti-miR-92 was significantly increased, demonstrating that miR-92 knockdown displayed a promoting effect on cell apoptosis (Fig.2C). We also noticed that the migration and invasion abilities of U251 and U87 cells were reduced distinctly when transfected with anti-miR-92 (Fig.2D and 2E). Collectively, miR-92 knockdown inhibited cell proliferation, migration and invasion capacities in glioma.

### 3.3 Neogenin was a target of miR-92

**Figure 3:**
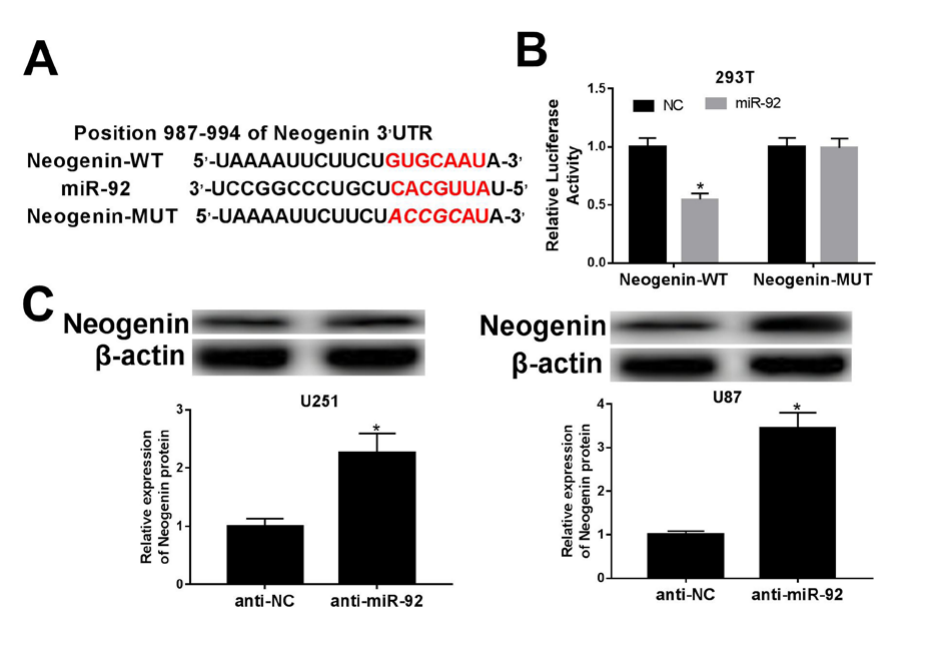
Neogenin directly interacted with miR-92. (A) The putative binding sites of miR-92 and neogenin were predicted by Bioinformatics analysis. (B) Luciferase activity was evaluated in 293T cells co-transfected with wild type of Neogenin 3’UTR (Neogenin-WT) or its mutant (Neogenin-MUT) and miR-92 mimic (miR-92) or its negative control (NC). (C) Neogenin protein level was examined by western blot assay in U251 and U87 cells transfected with miR-92 inhibitor (anti-miR-92) or its control (anti-NC). β-actin was applied as internal reference. *P < 0.05.

According to the bioinformatics analysis provided by TargetScan (http://www.targetscan.org), we found the potential binding site between neogenin and miR-92 (Fig.3A). To identify the prediction, 293T cells with dual-luciferase reporters were co-transfected with wild-type or mutant type of neogenin and miR-92 mimic or NC. The result revealed that luciferase activity of neogenin wild-type was conspicuously down-regulated in the presence of miR-92 (Fig.3B). Additionally, the protein expression of neogenin was dramatically escalated in U251 and U87 cells introduced with anti-miR-92 in comparison with anti-NC group (Fig.3C). Taken together, miR-92 could negatively regulate neogenin expression via target binding in glioma cells.

### 3.4 Neogenin suppressed the proliferation, migration, invasion and promoted apoptosis of glioma cells

**Figure 4:**
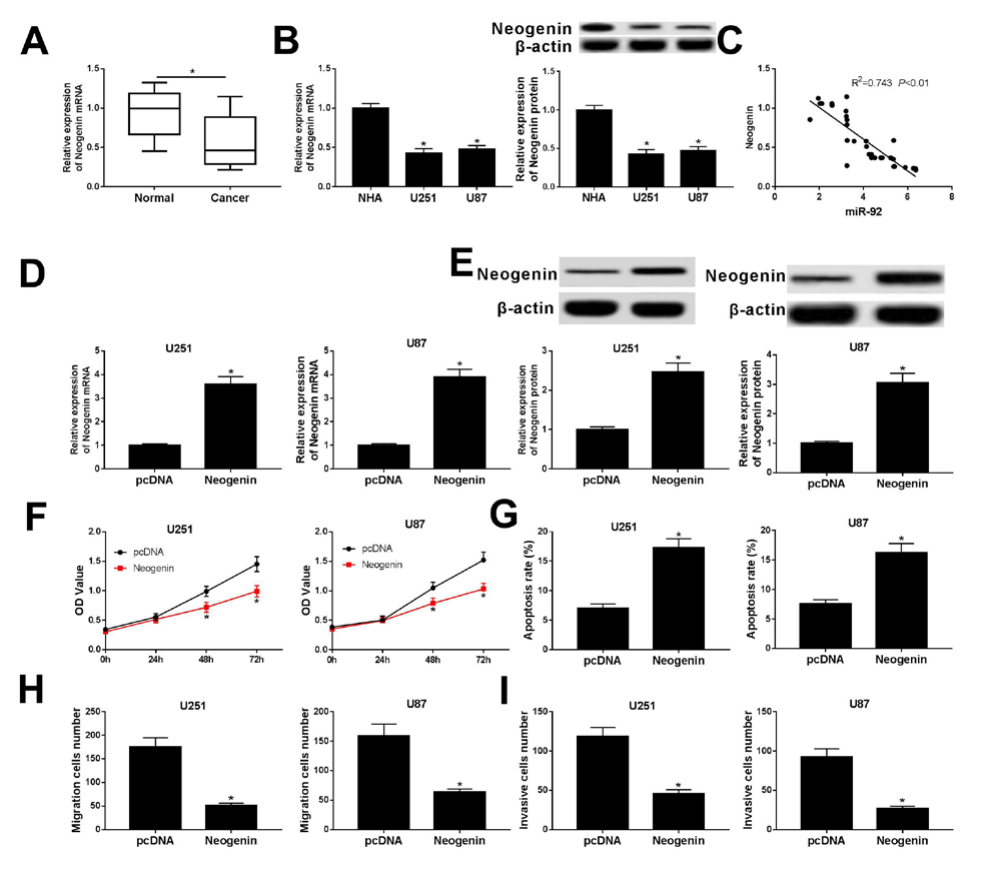
Neogenin modulated the proliferation, migration, invasion and apoptosis of glioma cells. (A and B) Expression level of Neogenin mRNA and protein was measured in glioma tissues and cell lines U251 and U87 by qRT-PCR and western blot assay, respectively. (C) Spearman’s rank correlation analysis testified the correlation between neogenin and miR-92 (R2=0.743, P< 0.01). (D-I) U251 and U87 cells were transfected with pcDNA-neogenin (Neogenin) or its empty vectors (pcDNA). (D and E) The neogenin mRNA and protein expression levels were evaluated by qRT-PCR and western-blot assay, respectively. (F) The cell proliferation ability was evaluated by MTT assay after transfection for different times (24 h, 48 h and 72 h). (G) Glioma cell apoptosis rate was detected after transfection for 24 h by flow cytometry. (H and I) The migration and invasion abilities of U251 and U87 cells were performed after transfection for 24 h by transwell assay. *P < 0.05.

Neogenin has been certified as a tumor suppressor and plays an essential role in regulating cancer cell progression and apoptosis. In order to assess the effects of neogenin on glioma cells, qRT-PCR was conducted to measure the expression of neogenin mRNA, and we discovered that the neogenin mRNA level was much lower in glioma tissue than the adjacent healthy tissue (Fig.4A). Similarly, the RNA and protein levels of neogenin were reduced in U251 and U87 cells as shown in Fig.4B. Moreover, we observed a noticeable inverse correlation between neogenin and miR-92 expression in glioma tissues (Fig.4C). Subsequently, U251 and U87 cells were forcibly expressing neogenin by transfection of pcDNA-neogenin vectors, and the expression and role of neogenin in glioma cells were figured out *in vitro*. Levels of neogenin mRNA and protein were unexpectedly up-regulated in pcDNA-neogenin-transfected cells (Fig.4D and 4E). Cell viability declined, and the apoptosis rate was elevated in neogenin-overexpressed U251 and U87 cells, as described by MTT and flow cytometry assays, demonstrating that neogenin could cause apoptosis and proliferation inhibition in U251 and U87 cells (Fig.4F and 4G). Moreover, the migration and invasion abilities of U251 and U87 cells were reduced sharply by neogenin overexpression in comparison with cells transfected with pcDNA (Fig.4H and 4I). All the data implied that neogenin inhibited proliferation, migration and invasion in glioma cells.

### 3.5 Neogenin mediated the tumor-suppressive role of miR-92 knockdown in glioma cells

**Figure 5:**
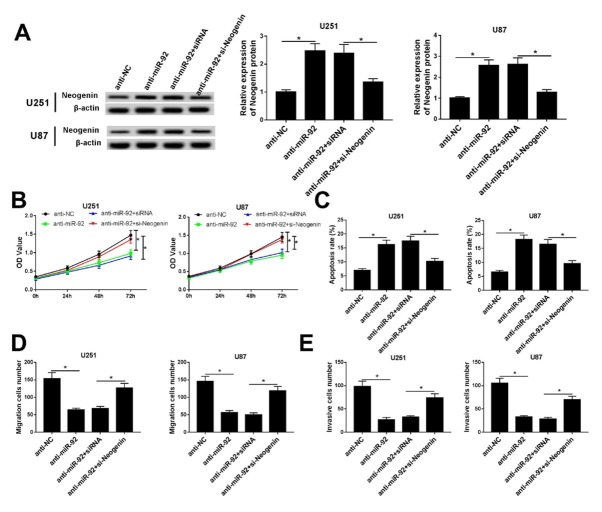
Neogenin upregulation mediated the protective effect of miR-92 on glioma cell proliferation, migration and invasion. U251 and U87 cells were transfected with anti-miR-92 or anti-NC, and co-transfected with anti-miR-92 and siRNA against neogenin (si-Neogenin) or its scrambled sequence (siRNA). (A) Expression level of neogenin protein was measured by western blot assay after transfection. (B) The cell proliferation ability was evaluated by MTT assay after transfection for different times (24 h, 48 h and 72 h). (C) Glioma cell apoptosis rate was detected after transfection for 24 h by flow cytometry. (D and E) The migration and invasion abilities of U251 and U87 cells were performed after transfection for 24 h by transwell assay. *P < 0.05.

Previous studies have clarified that neogenin participates in cell progression via miRNA regulation. To confirm the association between neogenin and miR-92 in regulating glioma cell progression, U251 and U87 cells were transfected with anti-NC, anti-miR-92, anti-miR-92+siRNA and anti-miR-92+si-neogenin. The neogenin protein was abundantly induced in U251 and U87 cells transfected with anti-miR-92 (Fig.5A). However, the upregulation of neogenin was significantly abolished by si-Neogenin. MTT and flow cytometry results exhibited the up-regulation of neogenin correlating with lower cell proliferation and a higher apoptosis rate (Fig.5B and 5C). Similarly, the migratory and invasive cells were dramatically decreased in U251 and U87 cells when neogenin was highly expressed (Fig.5D and 5E). Therefore, we concluded that neogenin upregulation could mediate the effects of miR-92 knockout on glioma cell proliferation, apoptosis, migration and invasion.

## 4 Discussion

In recent years, miRNA has developed to a hot topic in many pathological disorders, especially in cancer. It is well-documented that miRNA is capable of altering cellular metabolism by targeting a specific gene and further regulating its post-transcription [19, 20]. Besides, miRNA is essential in modulating cellular signal transduction, cell proliferation and differentiation, thereby the disturbance of miRNA can induce malignancies [21]. The discovery of certain miRNA can serve as diagnostic, prognostic markers and potential therapeutic targets in different tumorigenesis. Wei et al. found that the miR-509-3p level was up-regulated while the MAP3K8 level was down-regulated in polycystic ovary syndrome, and the interaction between miR-509-3p and MAP3K8 displayed instructional significance on regulating oestradiol production [22]. Accumulating evidence has identified that miRNAs are closely related with the regulatory network of brain cancer, for instance, miR-140-5p functioned as a suppressor to inhibit glioma cell proliferation and invasion whereas miR-363 displayed the opposite regulatory effects as oncogene [23, 24].

In our research, we have suggested that the up-regulation of miR-92/neogenin axis could contribute to glioma cell progression *in vitro* as evidenced by several findings. First of all, the expression level of miR-92 was largely up-regulated in glioma tissues and cell lines compared with the adjacent normal tissue and NHA, respectively. Furthermore, the elimination of miR-92 restricted cell proliferation, migration and invasion, and enhanced cell apoptosis inU251 and U87 cells. To expound the underlying molecular mechanism of miR-92 on glioma tumorigenesis and development, bioinformatics analysis online database TargetScan was recruited to predict the target gene, then we discovered miR-92 could bind to 3’-UTR neogenin specifically.

Neogenin, also known as a tumor suppressor protein Deleted in Colorectal Cancer (DCC), is low-expressed in many cancers, and this deficiency of neogenin increases the risk of tumor malignancy [25]. Previous studies have shown that an over-expression of neogenin could mitigate cell proliferation and induce programmed cell death; however, the deletion of neogenin showed the opposite effects [26, 27]. The role of neogenin relies on the ligand that binds to neogenin. For example, neogenin boosted cell adhesion when it bound to netrin; while neogenin stooged chemorepellant for cells when it was bound to repulsive guidance molecules. In cancers, neogenin exerts its biological activities through different mechanisms. Wei et al. proved that neogenin overexpression inhibited BMP-2-induced phosphorylation, thereby accelerating cell progression and reducing cell apoptosis in MDA-MB-231 breast cancer [28]. Inversely, Xueping Wang’s group observed that neogenin is over-expressed in gastric cancer, and neogenin could promote gastric cell adhesion by activating the Rac1/PI3K/AKT pathway [29]. To clarify whether neogenin could affect glioma cells positively or negatively, we detected the expression levels of neogenin mRNA and protein by qRT-PCR and western blot, then a reduction of the expression of both neogenin mRNA and protein was observed. In addition, the existence of neogenin inhibited cell growth, migration and invasion, as well as increased the apoptosis rate in glioma cells.

The main findings of our study include the following aspects. Firstly, excessive expression of miR-92 was detected in glioma tissue and cell lines. Nevertheless, miR-92 knockdown suppressed cell proliferation, migration and invasion, but increased the apoptosis rate in U251 and U87 cells, indicating miR-92 might play positive regulatory effects on glioma cell progression. According to bioinformatics prediction, there was a specific binding site between miR-92 and neogenin. Subsequently, we certified that miR-92 was inversely correlated with neogenin (R2=0.743, P＜0.01). Moreover, neogenin significantly inhibited cell growth and induced cell apoptosis by activating the cell death pathway, which was consistent with the previous report [30]. In order to confirm the regulatory effects of miR-92 and neogenin on glioma cells, we constructed anti-NC, anti-miR-92, anti-miR-92+siRNA and anti-miR-92+si-neogenin transfected glioma cells. The results showed that miR-92 knockout enhanced neogenin expression and further inhibited cell progression, whereas neogenin silence by transfection could rescue miR-92 knockdown-induced cell proliferation, migration and invasion. Collectively, our data demonstrated that miR-92 could regulate glioma cell progression by targeting neogenin.

## 5 Conclusion

Our results identified that miR-92 is a critical oncogene and plays an essential role in glioma cell growth, migration, invasion and apoptosis process. To the best of our knowledge, it is the first time that miR-92 has been discovered to alter glioma cell progression by directly targeting neogenin. The inhibition of miR-92 simultaneously up-regulated the neogenin protein expression level. Additionally, neogenin silence attenuated miR-92 mediated cell proliferation, migration and invasion. Therefore, miR-92 could serve as a potential diagnostic and prognostic marker in glioma patients.
